# Cholesterol-Dependent Serotonin Insertion Controlled by Gangliosides in Model Lipid Membranes

**DOI:** 10.3390/ijms251810194

**Published:** 2024-09-23

**Authors:** Jacques Fantini, Fodil Azzaz, Ryad Bennaï, Nouara Yahi, Henri Chahinian

**Affiliations:** Department of Biology, Faculty of Medicine, University of Aix-Marseille, INSERM UA16, 13015 Marseille, France; jacques.fantini@univ-amu.fr (J.F.); azzaz.fodil@gmail.com (F.A.); ryadbennai70@gmail.com (R.B.); henrichahinian@gmail.com (H.C.)

**Keywords:** serotonin, cholesterol, ganglioside, synapse, solubility, plasma membrane

## Abstract

Serotonin is distinct among synaptic neurotransmitters because it is amphipathic and released from synaptic vesicles at concentrations superior to its water solubility limit (270 mM in synaptic vesicles for a solubility limit of 110 mM). Hence, serotonin is mostly aggregated in the synaptic cleft, due to extensive aromatic stacking. This important characteristic has received scant attention, as most representations of the serotonergic synapse take as warranted that serotonin molecules are present as monomers after synaptic vesicle exocytosis. Using a combination of in silico and physicochemical approaches and a new experimental device mimicking synaptic conditions, we show that serotonin aggregates are efficiently dissolved by gangliosides (especially GM1) present in postsynaptic membranes. This initial interaction, driven by electrostatic forces, attracts serotonin from insoluble aggregates and resolves micelles into monomers. Serotonin also interacts with cholesterol via a set of CH-π and van der Waals interactions. Thus, gangliosides and cholesterol act together as a functional serotonin-collecting funnel on brain cell membranes. Based on this unique mode of interaction with postsynaptic membranes, we propose a new model of serotonergic transmission that takes into account the post-exocytosis solubilizing effect of gangliosides and cholesterol on serotonin aggregates.

## 1. Introduction

In the classic schematic neurotransmission conception, neurotransmitters are considered as perfectly soluble compounds in the aqueous inter-neuronal space, referred to as the synaptic space. This concept has prevailed because it is consistent with the required instantaneity of neurotransmission which is a discontinuous and iterative process. Hence, the solubility of neurotransmitters in water has been generally overlooked [1]. However, this parameter is critical as the behavior of neurotransmitters in water shows great variations according to their chemical structures. Indeed, a simple octanol/water partition analysis illustrates major differences among neurotransmitters [2]. Consequently, the kinetics of their diffusion in the synaptic cleft over a distance of 20–40 nm must necessarily differ, which in turn may impact the speed and the responsiveness of synaptic transmission. From this point of view, it is worth mentioning the neurotransmitter receptors classification proposed by Postila et al. (2016) [3] based on the behavior in water, highlighting the solubilization properties previously emphasized by Fantini and Yahi [4]. Overall, this classification is in agreement with the molecular view of the synapse developed by our group, considering water solubility as a critical parameter of neurotransmission function [4]. These studies are part of the broader framework of the role of membrane lipids in the regulation of synaptic transmission and brain functions [5,6,7,8,9,10,11,12]. Numerous studies have identified cholesterol as a major regulator of acetylcholine [13,14] and serotonin [11,15,16] receptors. Sphingolipids and in particular gangliosides then appeared as important partners of neurotransmitter receptors [17,18,19,20]. It is now generally accepted that lipid rafts, in which cholesterol and sphingolipids are concentrated, are signaling platforms playing a major role in synaptic transmission and in neurological disorders [21,22,23,24,25,26,27,28,29,30].

Serotonin is biosynthesized from tryptophan [31], an amino acid with a large aromatic structure which impairs its solubility in water. The metabolic pathway that transforms tryptophan into serotonin consists in a decarboxylation and the addition of an OH polar group [32]. At physiological pH, serotonin is positively charged [33], a property that, associated with the presence of the OH group, slightly improves its solubility in water compared to tryptophan (1.36 mg/mL for tryptophan vs. 2.5 mg/mL for serotonin) [34]. Nevertheless, the aromatic structure counterbalances the polar characteristic of the OH and ethylamine groups by conferring self-aggregative properties on the molecule through π-π stacking mechanisms which are particularly operative in synaptic vesicles [35] where neuronal serotonin concentration of several hundred of mmoles per liter has been measured (250–400 mM) [35]. It has been proposed that this aggregative state, which is in essence biologically inactive, instantaneously disappears after serotonin dilution to 10 mM, a concentration similar to the estimated biologically active serotonin in the aqueous synaptic space [36]. From simple electrostatic considerations, it could be reasonably argued that the cationic amine group of serotonin is transiently attracted by the electronegative carboxylate groups of gangliosides in postsynaptic membranes. The measurement of the bilayer electrostatic potential as a function of distance from the interface formed by ganglioside models as GM1, GD3 or GD3 lactone, in accordance with Gouy–Chapmann and Poisson–Boltzmann predictive theories, set limits beyond which the associated electric field has no influence on cationic neurotransmitter mobility [37]. In this respect, according to Juhola et al. [38], the attraction between cationic neurotransmitters and GM1 begins at a distance of 3.0–4.2 nm of the membrane midplane. This potential behavior can be correlated with the detection of a huge electric field at the synaptic space level. This local electric field arising around ionic current sources localized in a ganglioside rich environment (lipid raft microdomains), could reach 104–106 V/m [39,40] and consequently could improve neurotransmission efficiency by increasing the mobility of cationic serotonin by electro-osmotic drag. This electrostatic process could be accelerated by the dissolution of serotonin in the vicinity of postsynaptic membranes, extracting active monomers from inactive aggregates. Retrospectively, it is not surprising that gangliosides were the first receptors to be characterized for serotonin [41], even if these anionic glycolipids were then rather considered as electrostatic attachment sites for serotonin, optimizing serotonin concentration in the vicinity of postsynaptic receptors [42]. This scenario would suppose the formation of a serotonin–ganglioside complex of moderate affinity. The objective of the present study was to critically assess the role of raft lipids (gangliosides and cholesterol) in the bioavailability of serotonin in model membranes reflecting postsynaptic membrane microdomains. To this end, we used a combination of physicochemical and in silico approaches.

## 2. Results

### 2.1. Gangliosides Control the Dissolution of Synaptic Serotonin

**Figure 1:**
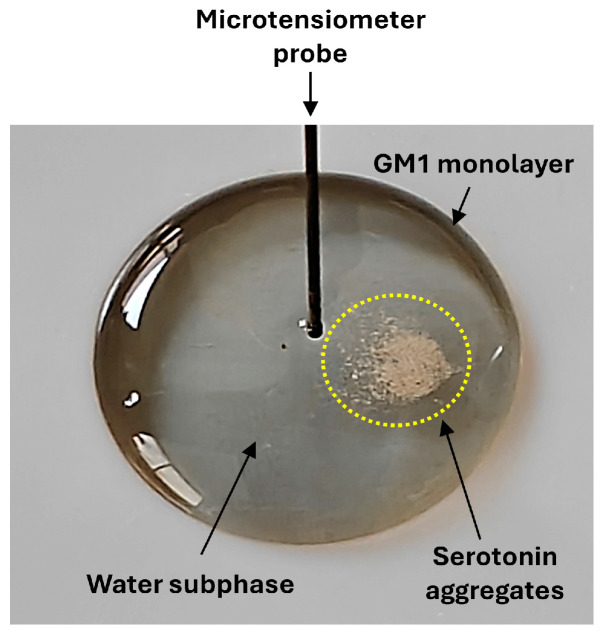
Experimental design. Non-HCl serotonin (140 mM) was injected into a 800 µL water drop. A lipid monolayer can be spread at the surface of the drop (e.g., GM1 gangliosides). Surface pressure measurements were recorded in real time with a platinum probe connected to a computer to assess the dissolution of serotonin aggregates by surface pressure measurements. Reflections from the laboratory windows are visible in the left part of the drop.

In a first series of experiments, we used Langmuir molecular monolayer technology to study the behavior of supersaturated solutions of serotonin. Our experimental setup was designed to record surface tension variation in real time with the potential to take snapshots of serotonin aggregates during the recordings. For these experiments, we used non-HCl serotonin, which has a limit of solubility in water similar to natural synaptic serotonin (110 mM). Serotonin was prepared at a stock concentration of 140 mM, and 16 microliters of this supersaturated solution were injected into a drop of water. Under these conditions, an insoluble fraction was clearly observable at the bottom of the well at the initial time of the experiment (Figure 1).

**Figure 2:**
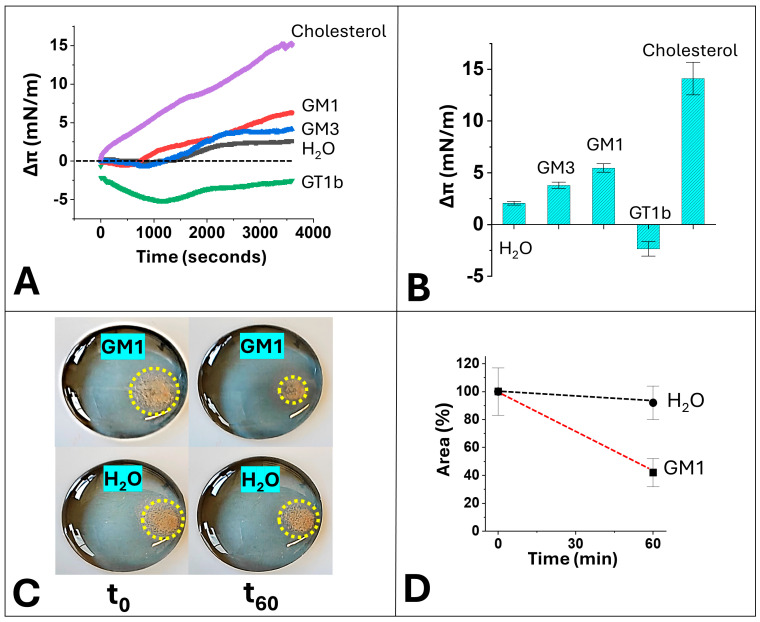
Selective effects of gangliosides and cholesterol on the dissolution of serotonin aggregates. The experimental design described in Figure 1 was used to measure the dissolution of serotonin aggregates in water or in presence of various monolayers of raft lipids. (**A**) Surface pressure measurements recorded in real time following the addition of serotonin. A representative curve is shown for each condition. (**B**) Statistical analysis of surface increase 1 h after the addition of aggregated serotonin in water under the indicated lipid (±SD, *n* = 3). All groups were statistically different (*p* < 0.05 in one-way analysis of variance and Tukey’s honest significant difference). (**C**) Snapshots of serotonin aggregates (circled in yellow) injected under a monolayer of GM1 or in water, at t_0_ or after 60 min of incubation (t_60_). (**D**) Mean areas of serotonin aggregates at t_0_ and t_60_ under a monolayer of GM1 or in water (±SD, *n* = 3).

The serotonin introduced into the drop forms a deposit clearly visible to the naked eye near the platinum probe, allowing the recordings of the microtensiometer. This device makes it possible to visualize the evolution of the serotonin deposit and to correlate it with surface pressure measurements (Figure 2). In pure water, these serotonin aggregates did not spontaneously disappear, and they remained almost unchanged after 1 h of incubation, resulting in very little variation in surface pressure (Figure 2A,B) and in the area occupied by insoluble serotonin (Figure 2C,D). The dissolution of the insoluble part of serotonin was then studied in the presence of a monolayer of cholesterol which appeared to be a good lipid attractor of serotonin molecules (Figure 2A,B). GM1 gangliosides spread on the surface of the water drop were also very efficient at inducing the dissolution of serotonin aggregates, as demonstrated by the progressive increase in the surface pressure of the ganglioside monolayer, due to the binding of serotonin molecules extracted from the aggregates (Figure 2A,B). This effect is accompanied by a clear decrease in the surface area occupied by aggregated serotonin, as illustrated in the snapshots of Figure 2C and the quantitative analyses of Figure 2D, with water being taken as a negative control. Interestingly, a similar (although weaker, but statistically significant over water) increase in surface pressure was observed with GM3 gangliosides, but not with GT1b gangliosides, showing that the phenomenon was highly specific (Figure 2A,B). These results validate the experimental system that we have developed to mimic the conditions of dissolution of serotonin in the synaptic space.

### 2.2. Molecular Dynamics Simulations of Serotonin Behavior with GM1, GM3 and GT1b Gangliosides in a POPC/Cholesterol Environment

**Figure 3:**
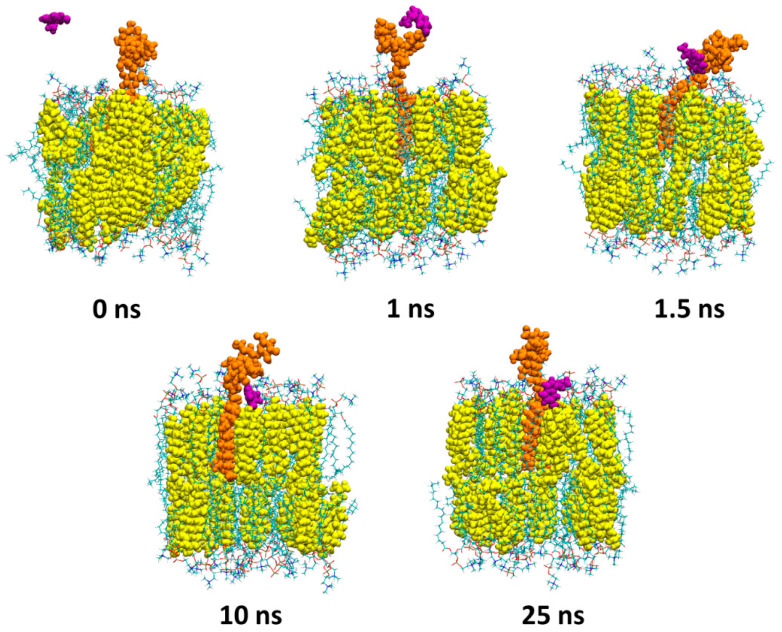
Molecular dynamics simulation of a serotonin molecule interacting with GM1. Snapshots were taken at 0, 1, 1.5, 10 and 25 ns. The serotonin is depicted as purple spheres (for visual clarity, only the serotonin that interacts with GM1 is shown), GM1 as orange spheres, cholesterol as yellow spheres and POPC molecules as lines colored by atom names.

To understand how serotonin enters into an interaction with GM1, GM3 and GT1b molecules at atomic resolution, we decided to run three different all-atom molecular dynamics simulations in which three serotonins were placed in the vicinity of GM1, GM3 or GT1b inserted into a POPC/cholesterol membrane at a molecular ratio of 1:1. For visual clarity, only the serotonin that directly interacts with ganglioside is shown. Figure 3 presents different snapshots corresponding to the simulation of serotonin with GM1. 

**Figure 4:**
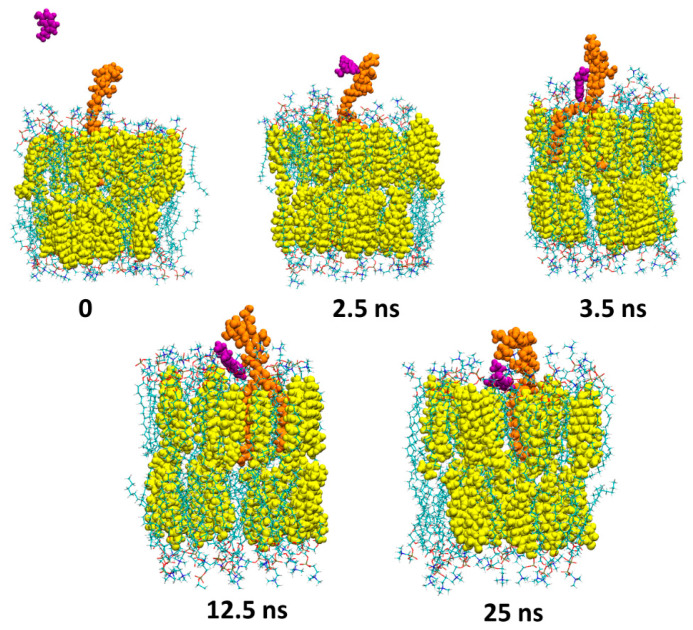
Molecular dynamics simulations of a serotonin molecule interacting with GM3. Snapshots were taken at 0, 2.5, 3.5, 12.5 and 25 ns. The serotonin is depicted as purple spheres (for visual clarity, only the serotonin that interacts with GM3 is shown), GM3 as orange spheres, cholesterol as yellow spheres and POPC molecules as lines colored by atom names.

The first image shows the initial configuration of the system. After only 1 ns, the serotonin (depicted as purple spheres) enters into contact with the polar part of GM1 (depicted as orange spheres) via electrostatic interaction between the cationic NH_3_^+^ group of serotonin and the anionic COO^−^ group of the sialic acid of GM1. Between 1 and 10 ns, the serotonin molecule is gradually sent towards the surface of the POPC/cholesterol membrane (POPC molecules are depicted as lines, while cholesterol molecules are depicted as yellow spheres) where it is maintained until the end of the simulation at 25 ns. Figure 4 presents snapshots corresponding to the simulation of serotonin with GM3. At 2.5 ns, the serotonin (depicted as purple spheres) enters into contact with the sugar molecules of GM3 (depicted as orange spheres). 

**Figure 5:**
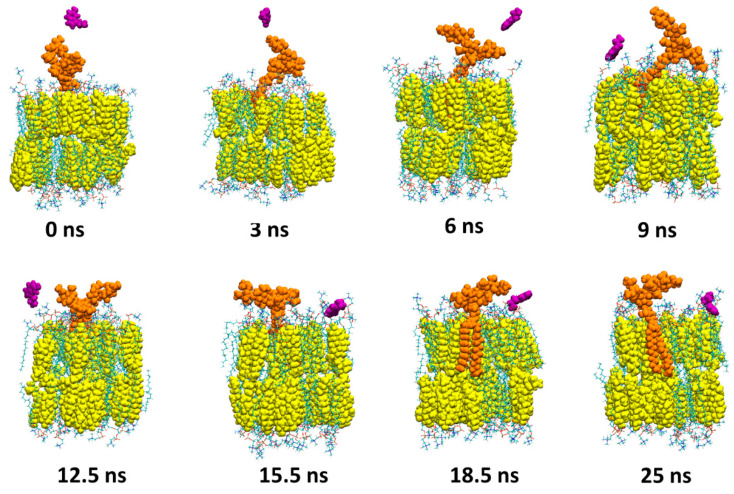
Molecular dynamics simulations of a serotonin molecule near GT1b. A total of 8 snapshots were taken for this figure representing the times 0, 3, 6, 9, 12.5, 15.5, 18.5 and 25 ns of the simulation. The serotonin is depicted as purple spheres (for visual clarity, only the serotonin that interacts with GT1b is shown), the GT1b molecules as orange spheres, POPC molecules as lines and cholesterol molecules as yellow spheres. Throughout the simulation, the serotonin molecule fails to interact durably with GT1b.

Between 2.5 and 12.5 ns, the serotonin molecule is gradually sent towards the surface of the POPC/cholesterol membrane (POPC molecules are depicted as lines, while cholesterols are depicted as yellow spheres) where it is maintained until the end of the simulation (25 ns). Finally, Figure 5 presents snapshots corresponding to the simulation of serotonin with GT1b. 

**Figure 6:**
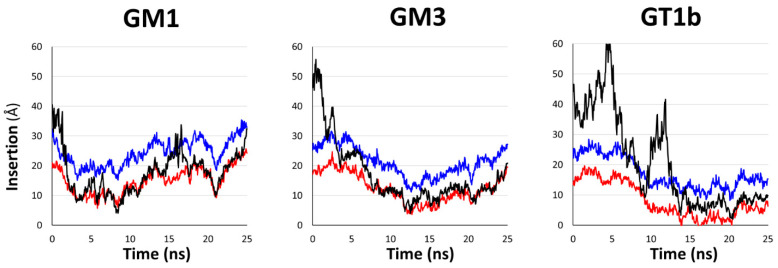
Graphs that show the insertion of serotonin in GM1 system (**left**), GM3 system (**middle**) and GT1b system (**right**). The black line corresponds to the mass center of serotonin, the blue line corresponds to the average protrusion of POPC molecules and the red line corresponds to the average protrusion of cholesterol molecules.

Unlike the simulations involving GM1 or GM3, the serotonin fails to interact durably with the sugar molecules of GT1b and the serotonin finally interacts with the surface of the POPC/cholesterol membrane from 15.5 ns until the end of the simulation. Each of these simulations shows that the intermolecular interactions between serotonin and the polar moiety of gangliosides are not durable, and in each case, the serotonin ultimately interacts with the surface of the membrane. Moreover, to gain more accuracy about the duration of contacts between serotonin and the membrane surface, we plotted the insertion of serotonin into the membrane surface over time. The plots in Figure 6 reveal that the insertion profile of serotonin is similar in GM1 and GM3 systems, while it is clearly different for GT1b. 

For GM1, as shown in Figure 6 (left plot), the insertion begins at 2 ns and lasts until the end of the trajectory. For GM3, the insertion begins at 3 ns and lasts until the end of the trajectory (Figure 6, middle plot). For GT1b, the insertion starts at 14 ns, and it is maintained until the end of the simulation (Figure 6, right plot). Altogether, our simulations are in good agreement with the experimental data by predicting that serotonin fails to interact in a stable manner to the sugar moiety of GT1b, while this molecule can bind the sugar moiety of GM1 or GM3.

### 2.3. Molecular Dynamics Simulations of Serotonin Behavior with POPC or POPC/Cholesterol in the Liquid Disordered Phase (Ld) of the Membrane

**Figure 7:**
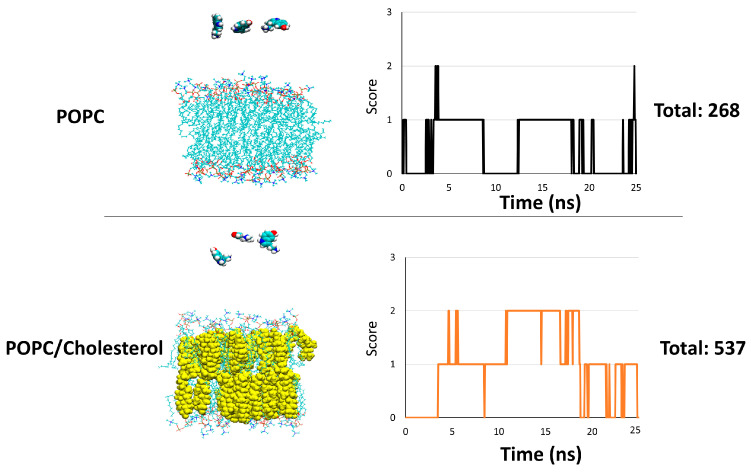
Score of serotonin in POPC (upper panel) versus POPC/cholesterol system (lower panel). A score of +1 is attributed when a serotonin is in contact with the surface of POPC or POPC/cholesterol membrane. The score was measured throughout the trajectory.

Our simulations of serotonin with gangliosides inserted into a POPC/cholesterol membrane showed that the serotonin always ultimately interacts with the surface of the membrane instead of adapting its conformation to interact durably with the polar moiety of ganglioside molecules. Based on these in silico observations, we wondered whether serotonin prefers to interact with POPC or cholesterol molecules. To answer this question, we performed two different simulations in which three serotonins are placed in the vicinity of a POPC or a POPC/cholesterol membrane at a molecular ratio 1:1. For each simulation, we plotted the “score” over time: a score of +1 is assigned when a serotonin molecule is interacting with the surface of the membrane. The plots are presented in Figure 7 (upper panel for POPC and lower panel for POPC/cholesterol). 

**Figure 8:**
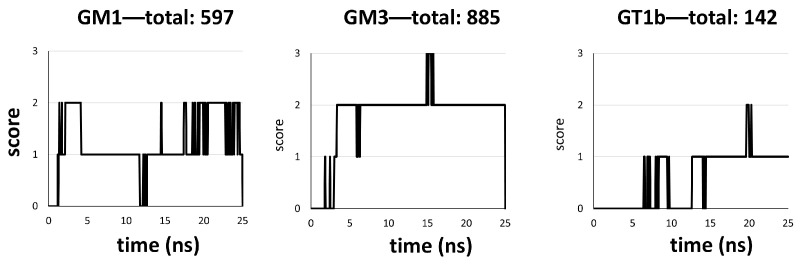
Score of serotonin in GM1 (**left**), GM3 (**middle**) or GT1b (**right**) systems. The score was obtained from the simulations presented in Figure 3, Figure 4 and Figure 5. A score of +1 is attributed when a serotonin is in contact with the surface of POPC or POPC/cholesterol membrane. The score was measured throughout the trajectory.

The analysis of the plots reveals that serotonin scored a total of 268 in the POPC environment against a total of 537 in the POPC/cholesterol environment. These in silico data suggest that the serotonin molecules interact more efficiently with the membrane surface when it contains cholesterol. The scores of the systems involving GM1, GM3 and GT1b are also plotted in Figure 8. The analysis of the graphs reveals that the serotonins were more in contact with the surface of the membrane in the GM3 system, with a total score of 885 (Figure 8, left plot), followed by the GM1 system, with a total score of 597 (Figure 8, middle plot), and finally the GT1b system, with a total score of 142 (Figure 8, right plot). We noted that the total score in the GT1b system was even lower than the POPC system, indicating that GT1b is not a good candidate to attract serotonin molecules towards the surface of the membrane. 

**Figure 9:**
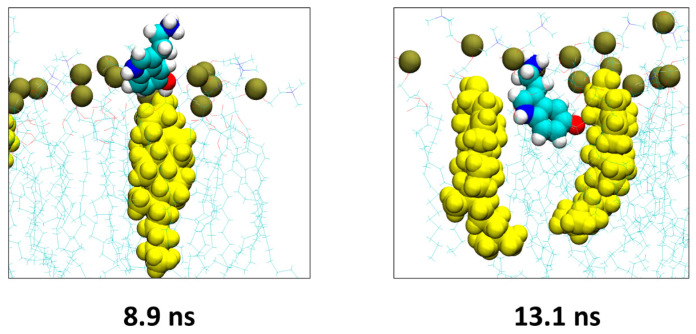
Representative example of the interactions between serotonin and cholesterol molecules. The snapshots were extracted from the POPC/cholesterol system presented in Figure 7. Serotonin is depicted as spheres colored according to atom name, cholesterol is depicted as yellow spheres, POPC molecules are depicted as lines colored according to atom names and the phosphorus atom of POPC is depicted as brown spheres.

A representative example of intermolecular contacts between a serotonin molecule and cholesterol is shown in Figure 9. At 8.9 ns, the serotonin molecule enters into contact with cholesterol via a hydrogen bond between the OH group of serotonin and the OH group of cholesterol. A bit later, at 13.1 ns, serotonin interacts with the apolar part of cholesterol molecules via a set of CH-π and van der Waals interactions.

### 2.4. The Initial Interactions between Serotonin and the Sugar Moiety of Gangliosides Initiate the Dissociation of a Serotonin Dimer

**Figure 10:**
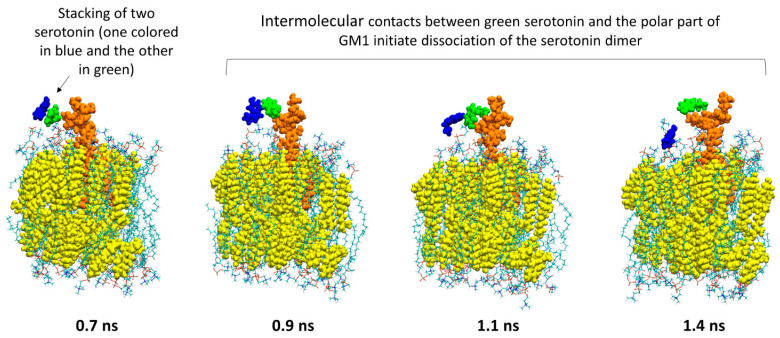
The polar part of GM1 initiates the dissociation of a serotonin dimer. The first image shows that a dimer of serotonin (one serotonin depicted as spheres colored in blue while the second one is depicted as spheres colored in green) is going to enter into an interaction with the polar part of GM1 (depicted as orange spheres). Then, the evolution of intermolecular interactions between the serotonin colored in green and the sugars of GM1 will cause the dissociation of the serotonin dimer.

**Figure 11:**
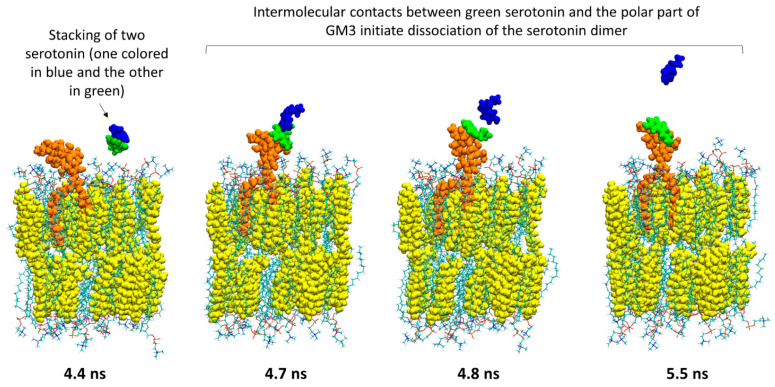
The polar part of GM3 initiates the dissociation of a serotonin dimer. The first image shows that a dimer of serotonin (one serotonin depicted as blue spheres and the other one as green spheres) is going to enter into an interaction with the polar part of GM3 (depicted as orange spheres). Then, the evolution of intermolecular interactions between the green serotonin and the sugars of GM3 will cause the dissociation of the serotonin dimer.

From a chemical perspective, the aromatic nature of the serotonin makes this molecule capable of oligomerizing via π-stacking. An interesting question to ask is “if serotonin molecules are released from the vesicles as large oligomers, which biomolecules could separate them?” Lipid raft gangliosides are obvious candidates. Figure 10 and Figure 11 present snapshots extracted from the simulations of serotonin—GM1 and serotonin—GM3, respectively (GT1b data are not presented since no interaction with serotonin occurred during the simulations). In both cases, the images show that the serotonin dimer (one serotonin colored in blue and the other colored in green) is dissociated by the intermolecular contacts established with the sugar moiety of GM1 (Figure 10) or GM3 (Figure 11). 

**Figure 12:**
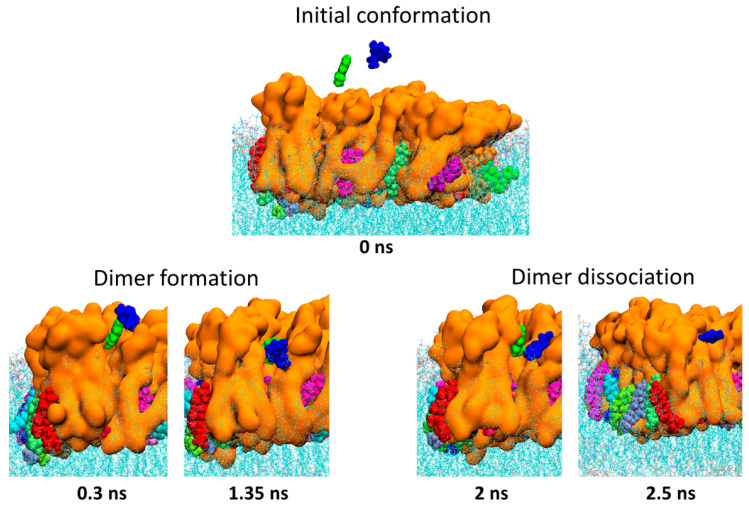
Dissociation of a serotonin dimer by a GM3 lipid raft. Snapshots were taken at 0, 0.3, 1.35, 2 and 2.5 ns. The GM3 gangliosides composing the lipid raft are depicted as an orange surface, the POPC molecules are depicted as lines colored according to atom names, the serotonin molecules are depicted as green or blue spheres and cholesterol is depicted as spheres (to improve clarity, each cholesterol is colored differently).

The same observation has been made in a GM3 lipid raft environment. As shown in Figure 12, the blue serotonin is attracted to the green serotonin, resulting in the formation of a dimer. Then, the evolution of the intermolecular interactions in the GM3 lipid raft leads to the dissociation of the serotonin dimer at 2 ns, as demonstrated by the exclusion of the blue serotonin out of the polar moiety of the lipid raft at 2.5 ns. 

Overall, these in silico data suggest that an isolated molecule of GM1 and GM3 can dissociate a serotonin dimer. Moreover, using the GM3 as a model to build a lipid raft structure, the GM3 lipid raft reproduces the same effect, which may suggest that this effect is not lost even if gangliosides are included in a lipid raft structure.

### 2.5. Serotonin Is a Surface-Active Compound, a Property That Gives Serotonergic Transmission Its Singularity

**Figure 13:**
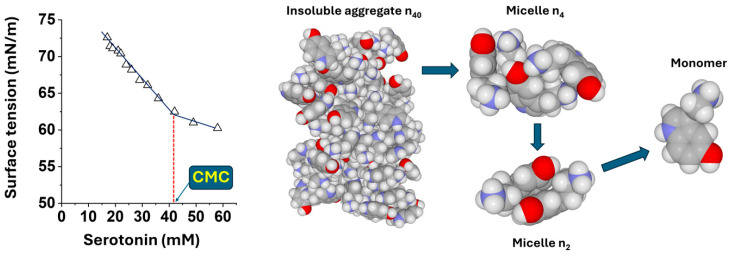
CMC determination (left panel) and molecular models of serotonin in various aggregation or monomeric forms (right panel). Serotonin micelles n_2_ and n_4_ are soluble and probably inactive in contrast with serotonin monomers. Oxygen atoms are colored in red, carbon in grey, hydrogen in white and nitrogen in blue. The red dash line indicates how the CMC is estimated (extrapolation of serotonin concentration at curve break).

Serotonin is an amphiphilic compound which confers on serotonergic transmission unique characteristics reflecting both its polar characteristic associated with its water solubility and its lipophilicity compatible with the intramembrane binding site in its protein receptors such as 7-TM GPCR 5HT-1A [43,44]. Like many amphiphilic compounds, serotonin has surface-active properties. In this study, we have determined its critical micellar concentration (CMC) by measuring the increased surface tension of water [45] in response to successive additions of water-soluble serotonin. Surface tension decreases when increasing the surfactant concentration up to the CMC, where the concentration dependence is nearly constant above the CMC (Figure 13). Under these conditions, the CMC of serotonin was estimated at 42 mM. Therefore, like many surface-active compounds, serotonin forms micelles in aqueous solutions. A serotonin micelle is a particle made up of a self-assembly of several molecules whose number depends on the serotonin concentration in water. A supersaturated serotonin solution represents a mixture of serotonin in several physical states, represented by monomeric serotonin, micellar serotonin and insoluble serotonin, in equilibrium (Figure 13). From a chemical point of view, the aromatic part of serotonin makes this neurotransmitter able to self-assemble via π-stacking. Our observations suggest that gangliosides (especially GM1) and cholesterol constitute the driving force for the dissolution of serotonin and its insertion into the postsynaptic membrane. 

## 3. Discussion

The identification of membrane lipids as privileged targets of serotonin confers on the membrane surface of postsynaptic neurons a functional serotonin storage capacity. This property could constitute an alternative mechanism for clearing the volume of serotoninergic synapse independently of receptors/carriers and it might be integrated into a global process of serotonergic neurotransmission [46]. In the present study, we developed a new experimental system that mimics the conditions of dissolution of serotonin in the synaptic space and allows real-time determinations of serotonin binding to lipids of the postsynaptic membrane. For this, we used non-HCl serotonin whose solubility limit is close to that of natural serotonin in the brain. We were able to follow the dissolution of serotonin aggregates by taking snapshots of the measuring drop of a microtensiometer at different times. This experimental system specifically designed for this study made it possible to measure in real time the impact of gangliosides and cholesterol on the dissolution of serotonin. We completed this analysis with molecular dynamics simulations taking into account water molecules and ions. This approach allowed us to follow the initial steps of serotonin interaction with gangliosides and cholesterol in their membrane environment. We also estimated the interaction energies of serotonin–ganglioside and serotonin–cholesterol complexes in these membrane systems. 

We showed that cholesterol and monosialylated gangliosides (GM1 and GM3) are able to extract and stabilize serotonin monomers from insoluble aggregates, whereas the trisialylated GT1b cannot (Figure 2). The higher efficiency of cholesterol compared with GM1 could be attributed to the respective molecular areas occupied by these lipids in a monolayer at an initial surface pressure of 20 mN·m^−1^ (i.e., under the experimental conditions of our study). Indeed, using molecular area estimations [47,48], we calculated that at this initial pressure, cholesterol molecules are 25-fold more numerous than GM1. 

The energy of interaction of the complex between serotonin and a model system with one ganglioside surrounded by two cholesterol molecules could be estimated at −54 kJ·mol^−1^ for GM1, −40 kJ·mol^−1^ for GM3 and −30 kJ·mol^−1^ for GT1b. Cholesterol alone was responsible for −32 kJ·mol^−1^. Moreover, the free energy value of cholesterol/serotonin association in a raft environment model (POPC/cholesterol/GM1) representing the liquid ordered (Lo) phase, and the free energy value of cholesterol/serotonin interaction in a membrane environment model representing the Ld phase (POPC/cholesterol) were of a similar order of magnitude (−32 kJ·mol^−1^). Thus, these estimations indicate that the affinity of serotonin is maximal for model membranes containing GM1, median for GM3 and quasi-null for GT1b, in full agreement with microtensiometry data (Figure 2). One may wonder why serotonin interacts favorably with GM1 and GM3 gangliosides but not with GT1b. If only the surface electrostatic potential is considered, the three sialic acids should confer a decisive advantage on GT1b over the other two monosyalylated gangliosides. However, it is clear that the ganglioside–serotonin interaction is more complex than a simple electrostatic attraction between an electronegative ganglioside and cationic serotonin. If this were the case, GM1 and GM3 gangliosides would have the same attractive effect on serotonin, which experimental data refute. Therefore, it seems that the glycan part of the gangliosides as a whole determines the mode of interaction with serotonin. It can be assumed that the ideal composition of this glycone part is achieved with GM1 (four sugars + one sialic acid), but only partially with GM3 (two sugars + one sialic acid). In the case of GT1b (four sugars + three sialic acids), the presence of three sialic acids must strongly increase the hydration and conformational flexibility of the ganglioside. Under our experimental conditions, this could limit any stable GT1b–serotonin interaction at the lipid–water interface due to the amphiphilic nature of serotonin. Further experiments would help to better understand these mechanisms.

In any case, it clearly appears that besides gangliosides GM1 and GM3, cholesterol may improve the insertion of serotonin whatever its localization in the plasma membrane, in the bulk membrane or in lipid rafts [49]. Thus, cholesterol can be considered as the lipid mediating the membrane insertion of serotonin, whereas gangliosides may rather control the initial electrostatic attraction of the neurotransmitter in lipid raft areas, as previously hypothesized [4]. Nevertheless, the ability of serotonin to interact with several types of membrane lipids [50,51] is probably due to its amphiphilic properties (Figure 13). In this respect, serotonin seems to accentuate the hydrophobic mismatch between lipid chains at the edge of vicinal phases, highlighting a serotonin-dependent alteration of the phase equilibrium by an increase in line tension at the periphery of membrane domains [46,50,51].

Based on our combination of atomistic simulations and monolayer experiments, we were able to distinguish three different physical states of serotonin in equilibrium: insoluble, micellar and monomeric serotonin (Figure 13). The kinetics of surface pressure measurements indicate that insoluble serotonin (Figure 1) does not dissolve instantaneously. The driving force of this slow process is dependent on GM1 gangliosides, which show the dissociating power of serotonin dimers (Figure 10, Figure 11 and Figure 12), and then of cholesterol, which maintains serotonin in the membrane (Figure 9). 

The serotonin/cholesterol complex characterized in the present study would allow one to optimize the permanent supply of serotonin to its receptor and/or transporter binding sites. As previously demonstrated for anandamide [52,53,54], cholesterol could be used as a membrane shuttle able to deliver serotonin to its protein targets by diffusion in the apolar part of the plasma membrane. Based on the data of the present study, we can hypothesize that gangliosides and cholesterol act together as a functional serotonin-collecting funnel. Correspondingly, a diffusible pool of serotonin bound to cholesterol might constitutively exist in serotonergic neuron membranes, allowing a timing offset between exocytosis and stimulation of postsynaptic membranes. This model is clearly distinct from the classic synaptic neurotransmission characterized by the switch on–off frequency of receptor-dependent signal transmission consecutive to exocytosis of synaptic vesicles. The ganglioside-dependent adsorption of serotonin monomers and the consecutive cholesterol-dependent membrane insertion of serotonin may constitute the driving force controlling the balance between the different forms of serotonin in the synaptic space and the complete dissolution of insoluble serotonin. This relatively slow process of synaptic serotonin would contribute to its long-lasting effect on the nervous system. 

## 4. Materials and Methods

### 4.1. Materials

Serotonin hydrochloride and non-HCl serotonin were purchased from Sigma-Aldrich (St. Louis, MO, USA). Lipids were purchased from Matreya. Ultrapure water was from VWR (Radnor, PA, USA).

### 4.2. Langmuir Microtensiometry

Serotonin–lipid interactions were studied with the Langmuir film balance technique using a Kibron Inc. (Helsinki, Finland) microtensiometer as previously described [55,56]. It is well established that this monolayer device is representative of a membrane bilayer and that it is one of the best experimental systems to measure lipid–protein interactions [57,58,59] and drug–membrane interactions [60,61] under perfectly controlled conditions. In these experiments, monolayers containing only the lipid of interest were analyzed to ensure that interactions were exclusively due to the binding of serotonin to the lipid studied. Monomolecular films of pure lipids were spread on pure water. After spreading of the film, 2 min was allowed for solvent evaporation. Serotonin was injected in the subphase (pH 7) with a P-100 Gilson pipetman (aggregated serotonin, kinetics experiments) or a 10 µL Hamilton syringe (serotonin hydrochloride, CMC experiment), and the surface pressure increases were continuously recorded as a function of time. The data were collected in real time with the FilmWareX program (Kibron Inc., Helsinki, Finland) and analyzed with one-way ANOVA and the Tukey’s test for post hoc. Snapshots of serotonin aggregates in the microtensiometer drop were taken with a OnePlus Open smartphone and directly imported into Microsoft PowerPoint without any image processing. These raw images are shown in Figure 1. Preliminary experiments were performed with NaCl 150 mM subphases instead of pure water, with similar results. 

### 4.3. Atomistic Molecular Modeling Simulations

The charmm topology and parameters of the serotonin were obtained using the tool “Ligand Designer” on Charmm-GUI (https://charmm-gui.org/ accessed on 30 May 2024). The initial coordinates of GM1, GM3 and GT1b were obtained using the tool “Glycolipid modeler” on Charmm-GUI. The POPC and POPC/Cholesterol membrane were obtained using the tool “membrane builder” on Charmm-GUI. Each system was solubilized using the tool “Add solvation box” and neutralized with Na+ and Cl^−^ counter ions at a final concentration of 0.15 mol/L using the tool “Add ions” in VMD. The systems were simulated (including the minimization and equilibrated runs) using the software NAMD 2.14 for Windows 10 coupled with the force field CHARMM36m (https://academiccharmm.org/ accessed 30 May 2024) at constant temperature (310 K) and constant pressure (1 atm). The cutoff of the calculation of non-covalent interaction was set at 12 A and a PME algorithm was used for the calculation of long-range electrostatic interaction in the periodic systems. 

The energy of the interaction of serotonin–lipid complexes was determined with the ligand inspector function of Molegro Molecular Viewer as previously described [56]. 

## 5. Conclusions

Our study reveals that gangliosides and cholesterol can exert a critical control on serotonergic transmission at two distinct levels: (i) gangliosides, especially GM1, catalyzes the extraction of serotonin monomers from insoluble aggregates present in the synaptic cleft, and (ii) cholesterol traps serotonin in the outer leaflet of the postsynaptic membrane, facilitating its transfer to serotonin receptors [4,42,62]. Moreover, raft lipids may exert broader effects on the clearance of serotonin from the synaptic cleft by controlling the synaptic concentration of serotonin and its reabsorption by vicinal brain cells. Based on the cholesterol-dependent insertion of serotonin in brain cell membranes, we propose a new model of serotonergic transmission that takes into account the presence of a constitutive pool of serotonin–cholesterol complexes that would allow long-lasting effects of serotonin disconnected from synaptic vesicle exocytosis. 

## Data Availability

The raw data supporting the conclusions of this article will be made available by the authors on request.
